# Scapular metastasis of hepatocellular carcinoma presenting as acute bleeding and hematoma: A case report of safe and effective treatment

**DOI:** 10.1097/MD.0000000000008736

**Published:** 2017-11-17

**Authors:** Ki-Hyun Kim, Hyung-Hoon Oh, Dong-Jun Son, Ji-Yoon Hong, Young-Hoon Jeong, Jin-Seong Jung, Hyeong-Min Yu, Dae-Seong Myung, Sung-Bum Cho, Wan-Sik Lee, Jin-Woong Kim, Young-Eun Joo

**Affiliations:** a Department of Internal Medicine; b Department of Radiology, Chonnam National University Medical School, Gwangju, Korea.

**Keywords:** bleeding, bone metastasis, hepatocellular carcinoma

## Abstract

**Rationale:**

The occurrence of bleeding and hematoma from bone metastasis of hepatocellular carcinoma (HCC) is extremely rare.

**Patient concerns:**

We present a case of scapular metastasis of HCC in a 69-year-old man who presented with acute bleeding and hematoma.

**Diagnoses:**

Chest computed tomography showed a large hematoma within the right pectoral muscle of the right upper chest and an exophytic metastatic mass in the right scapula with bony destruction, which caused the intramuscular hematoma. The final diagnosis was scapular metastasis of HCC presenting as acute bleeding and hematoma.

**Interventions:**

Selective right subclavian angiography showed a hypervascular metastatic lesion in the right scapula. Subsequently, embolization of the tumoral feeding artery using a microcoil was performed and tumoral bleeding was stopped.

**Outcomes:**

The patient was discharged on hospital day 14 without any complications.

**Lessons:**

Despite being extremely rare, the possibility of bleeding from bone metastasis of HCC needs to be considered. Transcatheter arterial embolization may be an effective means to treat bleeding from bone metastasis of HCC.

## 1 Introduction

Hepatocellular carcinoma (HCC) is one of the leading causes of cancer-related deaths worldwide, although its geographical distribution varies according to the prevalence of known etiologic factors, such as hepatitis B, hepatitis C, and alcohol use.^[1–3]^ Recent advances in both diagnostic and therapeutic techniques, such as multiphasic computed tomography (CT), magnetic resonance imaging (MRI), surgical resection, radiofrequency ablation, transcatheter arterial chemoembolization (TACE), and liver transplantation, have prolonged the survival of patients with HCC. However, extrahepatic metastasis of HCC, which has shortened patient survival, has been observed more frequently in recent years than in the past.^[1–3]^

The most common extrahepatic metastatic site of HCC is the lungs, followed by abdominal lymph nodes, bones, and adrenal glands. The common sites of bone metastasis include the vertebrae, pelvis, ribs, femur, scapula, skull, and sternum.^[4]^ Opioids, radiotherapy, and surgery are usually used for the treatment of bone metastasis of HCC.^[4]^

The occurrence of bleeding and hematoma from bone metastasis of HCC is extremely rare.^[5–14]^ Here, we present a case of scapular metastasis of HCC associated with acute bleeding and hematoma, and review the literature pertaining to this condition.

## 2 Case report

**Figure 1:**
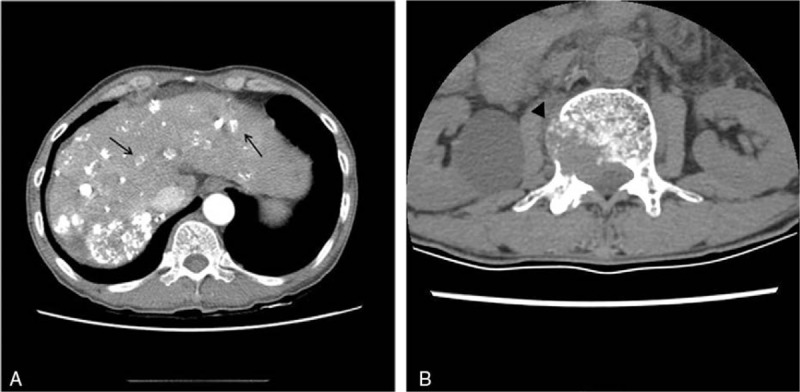
Computed tomography images of the abdomen and lumbar spine show multiple lipiodolized arterial enhancing hepatocellular carcinomas (arrow) in the cirrhotic liver after transcatheter arterial chemoembolization (A), and bony metastasis at the body of the second lumbar spine (arrowhead) (B).

**Figure 2:**
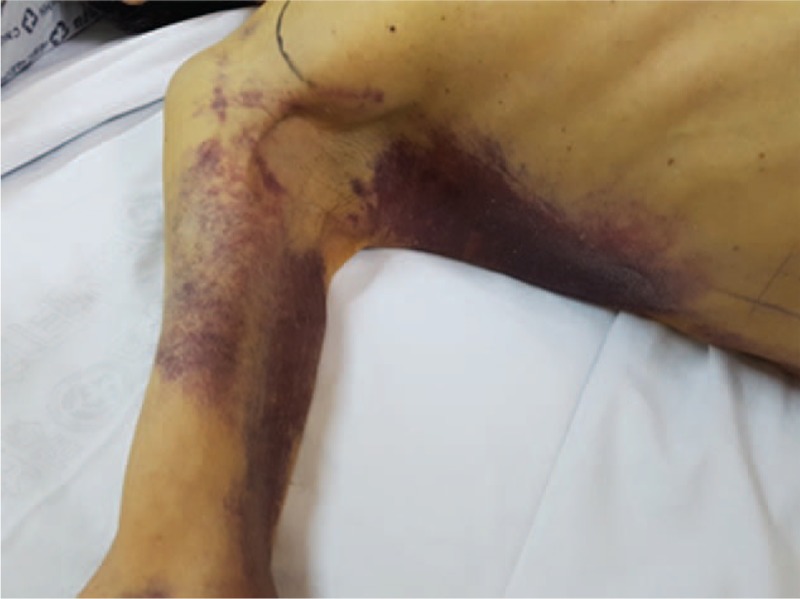
Physical examination reveals swelling and mild tenderness with skin color change in the right upper chest wall.

**Figure 3:**
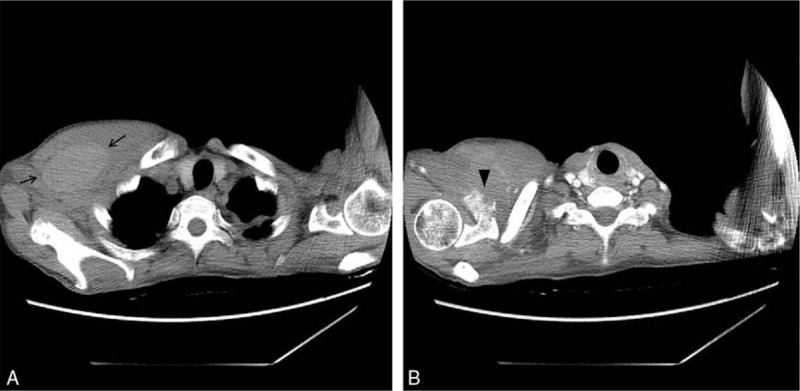
Chest computed tomography shows a highly attenuated large hematoma (arrow) within the right pectoral muscle of the right upper chest (A) and an ill-defined subtle enhancing exophytic metastatic mass in the right scapula with bony destruction (arrowhead), which caused the intramuscular hematoma (B).

**Figure 4:**
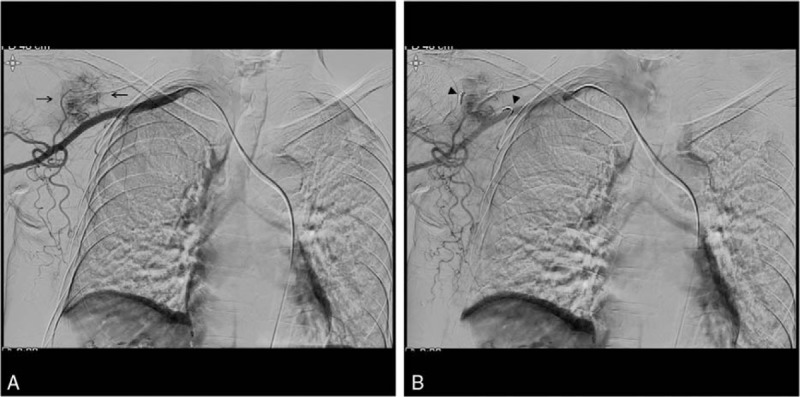
Selective right subclavian angiography shows a hypervascular metastatic lesion (arrows) in the right scapula (A). Subsequent embolization of the tumoral feeding artery using a microcoil (arrowhead) was performed (B).

A 69-year-old man was admitted to our hospital with a 1-month history of low back pain. He had suffered from HCC due to chronic hepatitis B for 4 years and had undergone TACE 4 times. On admission, laboratory examination revealed a white blood cell count 4700/mm^3^ (normal range, 6,000–10,000/mm^3^), hemoglobin 11.6 g/dL (normal range, 12–16 g/dL), platelet count 152,000/mm^3^ (normal range, 130,000–450,000/mm^3^), serum albumin 3.1 g/dL (normal range, 3.0–5.0 g/dL), aspartate aminotransferase 226 U/L (normal range, 5–37 U/L), alanine aminotransferase 55 U/L (normal range, 5–40 U/L), alkaline phosphatase 181 U/L (normal range, 39–117 U/L), and γ-glutamyltranspeptidase 324 U/L (normal range, 7–49 U/L). Total bilirubin was 2.3 mg/dL (normal range, 0.2–1.2 mg/dL) with 1.5 mg/dL direct fraction (normal range, 0.05–0.3 mg/dL), alpha-fetoprotein (AFP) 16,600 IU/mL (normal range, 0.74–7.29 IU/mL), and protein induced by vitamin K absence-2 (PIVKA-2) 6479 mAU/mL (normal range, 0–40 mAU/mL). CT of the abdomen and lumbar spine showed multiple lipiodolized arterial enhancing hepatocellular carcinomas in the cirrhotic liver after TACE, and bony metastasis at the body of the second lumbar spine (Fig. 1A–B). The following day, the patient complained of sudden pain in the right side of the chest. He denied any history of trauma or injury to that region. Physical examination revealed purpuric patches with swelling and mild tenderness with skin color change in the right upper chest wall (Fig. 2). Chest CT showed a highly attenuated large hematoma within the right pectoral muscle of the right upper chest, and an ill-defined subtle enhancing exophytic metastatic mass in the right scapula with bony destruction, which caused intramuscular hematoma (Fig. 3A–B). Angiography was performed owing to continued bleeding and increased hematoma despite medical therapy. Selective right subclavian angiography showed a hypervascular metastatic lesion in the right scapula. Subsequently, embolization of the tumoral feeding artery using a microcoil was performed and tumoral bleeding was stopped (Fig. 4A–B). The final diagnosis was scapular metastasis of hepatocellular carcinoma associated with acute bleeding and hematoma. The patient was discharged on hospital day 14 without any complications. At follow-up, he received radiotherapy for lumbar spine metastasis.

## 3 Methods

The patients provided signed, informed consent, and the study was approved by the Institutional Review Board of the Chonnam National University Hwasun Hospital

## 4 Discussion

Bone metastasis from HCC has been reported in approximately 1.6% to 16% of patients with HCC. The most common site of bone metastasis is the axial skeleton, such as the thoracic and lumbar vertebrae, followed by the pelvis, ribs, and long bones. Scapular metastasis from HCC is uncommon, and it occurs in approximately 3.8% to 7.8% of patients with HCC.^[4]^

Presenting symptoms from bone metastasis of HCC vary between localized pain, neurologic deficits, or pathologic facture, depending on the location and degree of the metastatic lesion.^[4]^ In addition, our patient complained of low back pain and sudden onset of right chest pain and hematoma, which was caused by lumbar and right scapular metastases.

HCC is often hypervascular and presents in combination with coagulopathy caused by underlying primary liver diseases, such as liver cirrhosis. It often leads to significant bleeding episodes, including variceal bleeding and hemoperitoneum, caused by rupture of varices and the primary liver tumor.^[1–3]^

Bone metastasis from HCC occurs via a critical step such as neoangiogenesis, which leads to migration and invasion of the cancer cells, a phenomenon similar to that observed in other cancers. Therefore, most bone metastases appear as hypervascular lesions implicating a possibility of bleeding. Moreover, the bone metastasis from HCC usually presents as osteolytic lesions on imaging studies. These osteolytic changes may lead to bone destruction with rupture of the vasculature in metastatic tissues.^[4–13]^ However, bleeding and hematoma from bone metastasis of HCC are extremely rare.^[4–13]^ Previously, cases of skull, rib, external auditory canal, maxilla, and mandible metastases from HCC with the development of bleeding and hematoma have been reported.^[5–13]^

In the present case, CT imaging and angiography showed osteolytic lesions with bone destruction in the lumbar spine and right scapula, and a hypervascular lesion in the right scapula, resulting in bleeding and hematoma.

The treatment options for bleeding from bone metastasis of HCC are manual compression; surgical hemostasis, including direct suture and resection of the lesion; radiotherapy; or transcatheter arterial embolization, according to the metastatic site and liver function status of the patients.^[4–14]^ In our case, selective right subclavian angiography and embolization of the tumoral feeding artery using a microcoil were performed and bleeding was stopped. To our knowledge, this is the first report on the treatment of bleeding from scapular metastasis of HCC by transcatheterarterial embolization.

In conclusion, bone metastases from HCC are typically hypervascular and osteolytic lesions. Therefore, despite being extremely rare, the possibility of bleeding from bone metastasis of HCC needs to be considered. Transcatheter arterial embolization may be an effective means to treat bleeding from bone metastasis of HCC.
